# Women’s experience of childbirth care in health facilities: a qualitative assessment of respectful maternity care in Afghanistan

**DOI:** 10.1186/s12884-023-06234-9

**Published:** 2024-01-10

**Authors:** Partamin Manalai, Nasratullah Ansari, Hannah Tappis, Young Mi Kim, Jelle Stekelenburg, Jos van Roosmalen, Sheena Currie

**Affiliations:** 1 Athena Institute, Vrije Universiteit, Amsterdam, Netherlands; 2 Jhpiego - Johns Hopkins University Affiliate, Baltimore, USA; 3 Department of Health Sciences, Global Health Unit, University Medical Centre Groningen, Groningen, Netherlands; 4 Independent Public Health Consultant, Amsterdam, Netherlands

**Keywords:** Respectful maternity care, Experience of care, Rights based care, Afghanistan

## Abstract

**Background:**

Respectful maternity care (RMC) remains a key challenge in Afghanistan, despite progress on improving maternal and newborn health during 2001—2021. A qualitative study was conducted in 2018 to provide evidence on the situation of RMC in health facilities in Afghanistan. The results are useful to inform strategies to provide RMC in Afghanistan in spite of the humanitarian crisis due to Taliban’s takeover in 2021.

**Methods:**

Focus group discussions were conducted with women (4 groups, 43 women) who had used health facilities for giving birth and with providers (4 groups, 21 providers) who worked in these health facilities. Twenty key informant interviews were conducted with health managers and health policy makers. Motivators for, deterrents from using, awareness about and experiences of maternity care in health facilities were explored.

**Results:**

Women gave birth in facilities for availability of maternity care and skilled providers, while various verbal and physical forms of mistreatment were identified as deterrents from facility use by women, providers and key informants. Low awareness, lack of resources and excessive workload were identified among the reasons for violation of RMC.

**Conclusion:**

Violation of RMC is unacceptable. Awareness of women and providers about the rights of women to respectful maternity care, training of providers on the subject, monitoring of care to prevent mistreatment, and conditioning any future technical and financial assistance to commitments to RMC is recommended.

**Supplementary Information:**

The online version contains supplementary material available at 10.1186/s12884-023-06234-9.

## Background

The collapse of the Afghanistan Government in 2021 and the loss of two decades’ worth of its achievements in addressing high levels of maternal and child mortality is disheartening. The very rights of Afghan women and babies to respectful quality maternity care is being denied by the devastating restrictions on women’s mobility, work and autonomy. Despite ongoing conflict and poverty, Afghanistan has made progress in provision of healthcare and maternal and child heath indicators since the Government was re-established in 2001 until its collapse at the hand of the Taliban in August 2021.

In 2004, a Basic Package of Health Services (BPHS) and an Essential Package of Hospital Services (EPHS) were designed and implemented at health system level by Non-governmental organizations (NGO) and Ministry of Public Health (MOPH) mainly with donor funds. These packages established district, regional and central hospitals, rural health clinics and a community health workers’ network [1, 2]. As a result, around 87% of the population had access to health facilities within two hours’ distance in 2018 [3]. To address shortage of skilled birth providers and in line with global recommendations midwifery education programs were supported technically and financially through national and international assistance. Skilled birth assistance in health facilities increased from 12.5% in 2003, to 34.3% in 2010, 51.0% 2015 and 58.8% in 2018 in health facilities. [4–7]. In addition, thousands of healthcare providers were trained in Emergency Obstetric and Newborn Care (EmONC) to improve quality of maternity and newborn care. Professional societies such as the Afghanistan Society of Obstetricians/Gynecologists (AFSOG) and Afghan Midwives Association (AMA), and accountability institutions including Afghanistan Nursing and Midwifery Council (ANMC) and Afghanistan Medical Council (AMC) were established to ensure quality and accountability of healthcare. These developments addressed bottlenecks to skilled birth care in terms of security, resources, capacities and coordination [8].

The World Health Organization’s (WHO) framework for quality of maternity and newborn care emphasizes that experience of care must receive equal attention as provision of care. In WHOs’ framework, effective communication, respect and preservation of dignity and emotional support are promoted to the level of evidence-based care, information system and referral system. Competent and able healthcare providers and the physical resources are expected to underpin both dimensions on equal grounds to attain the expected people-centered outcomes [9]. Women’s dissatisfactory experiences in the health system, spanning from lack of access to care, to improper examinations, neglect and carelessness, verbal abuse and shouting, discrimination especially against the poor, affect their ability to access healthcare and navigate the system [10].

Bohren’s 2015 systematic review of mistreatment identifies physical, sexual and verbal abuse, stigma and discrimination, substandard care, poor provider-recipient rapport and health system conditions and constraints [11]. The increasing acknowledgment of rights-based care informed the updated Charter by the White Ribbon Alliance (WRA) on the Universal Rights of women and newborns in their entitlement to 1) freedom from harm and ill-treatment; 2) right to information, informed consent, respect to choices and preferences; 3) privacy and confidentiality; 4) (newborn’s) dignity and respect; 5) equality and equity; 6) healthcare and health; 7) liberty, autonomy, self-determination and freedom; 8) (children's) being with their parents or guardians; 9) (children's) identity and nationality from birth; and 10) adequate nutrition and clean water [12].

Lack of RMC destroys women’s’ trust and leads to reduction of facility-based births and promotion of homebirths not assisted by skilled professionals. The result is increased risk of maternal and newborn morbidity and mortality and adverse mental health consequences [13]. The National Strategy for Reproductive, Maternal Newborn and Adolescent Health (RMNCAH) of Afghanistan under the previous Government committed to RMC, along with other related interventions such as increasing the number of providers, improving their compensation, capacity building and mentorship initiatives [14]. Despite these achievements, RMC remains a challenge and little research data are available on this important component in Afghanistan. The national 2016 assessment of quality of maternal and newborn care in Afghanistan showed that as low as 40.1% of women received an explanation about care on admission in labour, 34.3% were told what will happen during giving birth and 37.1% were encouraged to walk and assume different birthing positions [15]. Healthcare providers admitted to neglect, verbal abuse, demanding bribes and shouting at women which they blamed on excessive workload, poor compensation and injustice in recognition of their hard work [16]. Data from other countries are indicating mistreatment in maternity care in a subtler way. In Ghana, Guinea, Myanmar and Nigeria, 41.6% of observed women and 35.4% of interviewed women reported physical or verbal abuse, or stigma or discrimination [17]. In Eastern Mediterranean countries prevalence of physical abuse ranged from 6.5% to 18.6%, non-consented care from 35 to 100% and breach of confidentiality from 5.6% to 40%; while other forms of mistreatment including violation of dignity, discrimination, detention and abandonment were also found widespread. [18]. A survey of USAID’s Maternal and Child Health Integrated Program (MCHIP) projects included respondents from Africa, Latin America and Asia who reported lack of privacy in 56% of the women, in 50% harmful practices, lack of information, lack of informed consent and lack of choice of birthing position, while 46% reported verbal abuse and 42% lack of companion, abandonment, lack of confidentiality, and being denied food and drinks [19].

Lack of in-depth understanding of RMC in Afghanistan led to the Ministry of Public Health (MoPH) and its partners to conduct a qualitative study of expectations and experiences of childbirth in health facilities. The aim of this study was: 1) to understand women’s perceptions of maternity and newborn care, 2) healthcare providers’ perceptions of women’s rights to respectful care and drivers for their mistreatment and 3) the perceptions of selected health care managers and policy makers. This enquiry into RMC during childbirth triangulated the findings from women who experienced childbirth care in health facilities, healthcare providers who assisted women during childbirth and healthcare managers and policy makers.

The study was expected to guide the MoPH and partners to make maternity care more acceptable to women. Afghanistan is currently suffering escalation in insecurity, socioeconomic pressure, psychological stress and systematic discrimination against women including midwives and obstetricians.. During two decades of progress before the collapse of Government in 2021, women’s access to healthcare had increased considerably. Assessment of women’s expectations and experiences of care during childbirth revealed, however, many gaps in RMC that demanded deliberate and focused attention. It is important to maintain women’s access to healthcare in the current situation. Respectfulness of healthcare is equally imperative in the face of Taliban’s discriminating attitude towards women. Presenting the evidence on women’s expectations and experiences of care and the proposed solutions are therefore extremely important, relevant and timely. This will certainly enhance awareness and sensitivity of the national and global community to press for respectful, quality maternity care in Afghanistan.

## Methods

This study was carried out in public health facilities with three key stakeholders including: 1) women who used maternity care in health facilities, 2) health care providers who were involved in providing maternity care and 3) key informants representing healthcare managers and policy makers. Focus group discussions (FGD) were carried out with the women who used maternity care and the healthcare providers. For these two categories, FGDs were preferred to in-depth interviews as the group dynamics would facilitate an open and freer discussion in what is predominantly an oral culture where women may feel less confident about expressing views in a one to one situation. In-depth interviews were conducted with healthcare managers and policy makers considered as key informants for the study (key informant interview (KII)). A purposeful sample was selected including one facility from four most populated areas of Balkh, Kandahar, Herat and Nangarhar provinces. Within each province one District Hospital (DH) was selected randomly, excluding provincial capitals to evade the complexity of large provincial hospitals.

Women who had given birth in these facilities during the previous 12 months were selected randomly from the facility register books for women’s FGDs. Providers of maternity care from the same facilities were included for provider FGDs. Key informants from the same provincial centers as well as from MoPH participated in KIIs.

The study tools were semi-structured discussion guides based on a previous WHO study with similar objectives [20, 21]. These were translated into local languages. The tools were tested for the Afghanistan context, using mock interviews and FGDs with female Jhpiego staff with midwifery and obstetric clinical backgrounds as intensified insecurity at the time of the study did not allow field testing with external candidates from outside the office.

Per socio-cultural requirements, the field study team comprised of four female researchers with previous experience in conducting FGDs and KIIs. The team spoke three local languages, cumulatively spoken by all target participants. Verbal consent from each participant was obtained. The researchers were trained for three days in study methods and use of the tools. Each FGD was facilitated by two researchers using written and/or recorded responses as permitted by the participants. The same teams also conducted KIIs. KIIs were recorded and written notes of researchers’ observations were also taken. All FGDs and KIIs were conducted in person.

Thematic analysis of the notes and transcribed recordings was performed [22]. Themes, initially deduced from the study objectives and the items from the study tools, were adapted to the emerging patterns during data analysis. Coded textual data were analyzed with emphasis on both manifest and latent contents. Excerpts of the findings were regularly translated to English and examined by the PI and other contributors for their inputs. During the analysis, in-country and online meetings were held with co-investigators to consider issues of reflexivity including examples of mistreatment which can be normalized in Afghanistan and possibly missed in analysis, as well as cultural and gender norms. Transcripts produced by the four field researchers were reviewed independently by men and women of the whole research team as well as jointly by both men and women team members to ensure gender perspectives and context in data interpretation. Analysis was performed using MS Word to ensure close contact and interaction with the data by all members of the team.

### Findings

A total of four FGDs with 43 women who had given birth, four FGDs with 21 health care providers and 20 KIIs with key-informants were completed (Table 1).

**Table 1:** Numbers of FGDs and KIIs with participants

Data collection methods	# sessions	# Participants	# Participants per FGD
A. FGDs with groups of women who had given birth in the selected facilities in the past 12 months	4	43	9 – 12 (average 11)
B. Focus group discussions with groups of healthcare providers (all female)	4	21 (15 midwives, 5 obstetrician/gynecologists, 1 nurse)	5 – 6 (average 5)
C. In-depth interviews with key informants	20	12 from MoPH 7 from BPHS implementing NGOs 1 from another ministry (not disclosed for anonymity)	

Key themes that emerged from data analysis included: 1) perspectives on motivators and deterrents when using health facilities for childbirth; 2) accounts and experiences while using health care from providers, and 3) awareness of rights and suggestions for improvement of care. Both women and providers reflected on out-of-pocket payments as barriers for using maternity care. Providers and key informants also included workplace respectfulness (Table 2).

**Table 2:** Emerging themes from the analysis

FGDs with women who had given birth
• Motivators for use of health facilities • Deterrents for use of health facilities • Experiences of providers’ behavior (including adequate and respectful treatment and mistreatment) • Expected behavior of maternity care providers • Out-of-pocket payments • Awareness of rights-based RMC • Suggestion for improvement of maternity care
**FGDs with maternity care providers**
• Motivators for use of health facilities • Deterrents for use of health facilities • Providers’ narratives of maternity care including quality of care and presence of mistreatment and abuse • Providers’ experiences of out-of-pocket payment for maternity care • Awareness of rights-based RMC • Suggestions for improvement of maternity care • Experiences and expectations for respectful workplace environment
**In-depth interviews with key informants**
• Awareness of rights-based RMC • Key informants’ narratives of maternity care including quality of care and presence of mistreatment and abuse • Suggestion for improvement of maternity care and ensuring respectful care • Experiences and expectations for respectful workplace environment

### A- Women´s perspectives

#### Motivators for using health facilities for childbirth

Availability of medicine, care for complications such as bleeding, high or low blood pressure, malpresentation and malpositions and post abortion care were cited as advantages of care seeking in health facilities, especially if the needs arose during nighttime. A key motivator for using health facilities for giving birth was women’s trust in facilities with knowledgeable, caring and respectful providers, consistently mentioned in FGDs. Other reasons included the cleanliness of the facility as compared to their homes, availability of free care in public hospitals, and the stereotype of illiterate women giving birth at home. The decision makers about seeking care were the husbands or mothers-in-law in most instances rather than the women themselves. One woman mentioned:

*“Well, God forbid, if the baby is in wrong position, or bleeding happens, or [baby] is weak, there are midwives in the clinic and they will give you injections or pills or they will send you to another hospital for treatment.”*

### Deterrents from using health facilities for childbirth

Participants preferred to refer to “other women”, who do not use health facilities rather than their own preference. Reasons enumerated by women included stigma and shame of being exposed to strangers even other females, financial barriers and bribes, dissatisfaction during previous experiences with inadequate attention and mistreatment, resistance of husbands for cultural reasons and perceptions of poor quality of care in facilities. Remoteness of facilities and security barriers, especially at night, were also mentioned.

*“Old people [women] told us we always delivered at home, you youngsters are weak and afraid of giving birth. … [therefore in past] I was ashamed of the midwives… but when I came, it was a big comfort to deliver in health facility.”*

*“Some [people] have no money to come to clinic; they have to come from a large distance”.*

*“Women want to come to hospital [for giving birth] but husbands think it is a shame”.*

#### Experiences of providers’ behavior (including adequate and respectful treatment and mistreatment)

Participants mostly named positive behaviors of providers and acceptable care with “clean, safe and secure birth rooms, clean towels and clothes, serum, injections, blood pressure checks and prescription of medicine”. Privacy, presence of healthcare providers at all times, access to a female companion and ability to move around were mentioned. Providers’ behavior was illustrated mostly as “caring” and “supportive”, but with "some delays". However, instances of misbehavior and discrimination against the poor, nepotism, shouting, scolding and denial of care to some women and complaints of dirty floors and no heating were also heard.

*“They [healthcare provider] stay with me, gave me serum and injections, checked my blood pressure. They looked at their watch and when the time was up they told me to push…finally the baby came, they put the baby on my belly… they watched me and my baby for six hours…then they took my phone number and said she will call me sometimes….if anything happens call me or come back*”.

*“Of course they treated me badly, if they didn’t why they didn’t help me [ignored me]? They discriminate between wealthy and poor. They accuse us that we linger in the facility unnecessarily and we try to find excuses to stay in hospital”.*

Participants reported about “others” rather than recounting their own experiences of mistreatment in the facilities. Physical mistreatment such as shouting and slapping or verbal abuse such as reprimanding for getting pregnant were mentioned.

*“One woman came from very far away, and she gave birth in the car. But her placenta was not delivered at the time of her arrival to the hospital. Hospital midwives helped us to deliver our babies but none of them helped that woman because they were angry - why did the woman deliver in the car?”*

*“Midwife does not understand the pain that we have in giving birth. She used her hand to pick up the newborn and hit and shouted at me to spread my legs”.*

Some women were sympathetic with over-burdened providers, who also had anxiety due to insecurity in health facilities, to excuse their mistreatment of women, but also pointed to harsh personality and lack of skills and experience of providers. Issues like lack of equipment, supplies and amenities were not mentioned by the women. However, no one considered any level of misbehavior to be acceptable. A few women mentioned providers constrained the woman by holding her down during birth, but considered it as acceptable for their own safety.

### Expected behavior of care providers

Women expressed their trust when the midwives provided the care they needed, but also indicated they could have been “more helpful”, indicating their latent dissatisfaction. Agility in attending and talking to the women as well as reassurance were emphasized. They expect providers to let their companions remain at the bedside throughout childbirth, but they were usually led out of the room just as birth commenced. They did, however, not indicate they were in a position to change the decision of the providers in such matters.

*“They [providers] help, when you have pain they help, you feel reassured that here you have a midwife and she will help if anything happens”*

*“A doctor should have good behavior, should give us good morale during giving birth, should not reprimand us for anything.”*

### Out-of-pocket payments

Women who gave birth, have given gifts of 50 to 500 Afghanis (~ USD 0.6 – 6) to cleaners and providers especially if the newborn was a boy to ensure their continued attention. They also complained that in spite of the policy of free medicine in public facilities, they had to buy medicine from drug stores and that the prices in private facilities are too high for them.

A woman said:

*“This time, the doctors told me that I will need Cesarean [section], and when I delivered normally they told me I should give them “treat” [gift in cash] so I give them five hundred Afghani as gift.”*

*“My husband’s sister gave birth here, he was a boy, the doctor asked for 500 Afghani, we gave her money. Everybody asks for presents; the cleaner asks for presents as well”.*

### Awareness of rights-based RMC

The majority of the participants were illiterate and did not know about the MoPH’s Patient’s Charter of Rights, although all were aware of their right to RMC, to be given free in public facilities with access to “good” doctors and midwives, ambulance transport and quality medicine.

*We have the right, that we come here at the time of illness [giving birth], we must be treated here, this is our right.*

*“It is written that everything is free, so we come, if it were for money, we would not come”*

### Suggestions for improvement of maternity care

Participants indicated that care would be more acceptable if there were more labour rooms and beds in existing health facilities, if companions were allowed to stay with them till completion of giving birth, if all facilities had ultrasound and providers avoid speaking harshly to women during birth.

*Number of [birth] rooms need to be increased, birth rooms should be warm, in the summer [rooms] should be cool.*

*“One woman said that electricity should come, another one that ultrasound should come”.*

### B- Providers’ perspectives

#### Motivators for use of health facility

Providers indicated that people chose facility birth for care when complications such as preeclampsia/eclampsia, retained placenta, shock and bleeding occur as well as for access to vaccines, blood transfusion, RhD immunoglobulin injection, good equipment and supportive care givers. They believed women who came to the facility for antenatal care would also come for childbirth.

*“Death and life is in the hand of Allah, however, every woman wants to give birth in a clean and safe place where skilled birth attendant is available to provide all of the care”.*

#### Deterrents for use of health facilities

Factors preventing women giving birth in health facilities, mentioned by providers, were traditions and family restrictions, preference for birth assistance from ‘dais’ (local word for traditional birth assistants) and relatives, insecurity during the night, unavailability of providers at the moment of attendance, fear of mistreatment by the providers, high transport costs, costs incurred in the health facility and lack of awareness of the people about availability of maternity care. Providers stated that the decision to use facilities for maternity care is taken by the husband, mother-in-law, father-in-law or other elders in the family.

“*Just because of this poverty they can’t come to facility. Some [of them] come from a distant [place] and if they want to come they don’t have the money for transport. In the middle of the night, a car will ask at least two thousand Afghanis. Also the people’s level of knowledge is low, and there is insecurity too.”*

### Providers’ narrative of maternity care including quality of care and presence of mistreatment

Providers insisted that the women receive all necessary care items in a clean, safe and confidential environment. Freedom to move around while in labor, choosing preferred birthing positions, continued presence of provider with the women, allowing women to have a companion till the time of giving birth, reassurance of mother and informing her of the progress of labour were enumerated. Providers insisted women received the necessary care such as promoting skin-to-skin contact of the newborn with the mother, vaccination, counselling on nutrition and hygiene. Providers reported that women insisted their in-laws leave at the time of giving birth. Some upright birthing positions were rejected as unclean and causing complications. Providers reported instances of delays in care and unnecessary induction or augmentation to speed up childbirth. They insisted on moral support, privacy and confidentiality, conversing on labour progress and baby’s condition including gender, using women’s name and speaking their language.

*“Our birth room is clean and we have a separate postpartum room with two beds. The door has a curtain, we change the bedsheets and allow the woman to give birth the way she likes. Some women even don’t allow their mother, but if she likes we allow companions to stay with her during giving birth. Some husbands bring their wives on a bike, so we have to deliver her alone without someone from her family. Women can walk, eat, drink as they like until the cervix is dilated enough. We insist that women should give birth on the birth table because it is clean, but if the woman cries and makes noise [insists very much], we spread a plastic sheet on the floor and allow her to give birth on the ground.”*

Providers reported instances of mistreatment and abuse such as beating, slapping, insulting, shouting, reprimanding and scolding for bad hygiene, denying taking blood pressure or other care items, but these were attributed to the personal character of individuals and colleagues’ exhaustion. Conversely the providers indicated verbal and physical mistreatment of providers by some women. Reasons mentioned for mistreatment included small birth rooms, insufficient equipment and supplies, inadequate coaching and support and excessive work burden on providers. They disapproved of the mistreatment and insisted addressing the causal factors would make maternity care more respectful.

### Providers’ experience of out-of-pocket payment for maternity care

Providers denied unanimously receiving any money from women in public facilities, but did admit to small gifts given voluntarily to them. They insisted no one was denied care for not paying.

*“The other day, a woman had a boy after four daughters. Next day she sent me some dried spinach and insisted that I should take it. If I did not take it she would be offended.”*

*Some women give us 100 or 200 Afghani when their baby is born, God forbid, we never ask for money. People are very poor, very poor”.*

### Awareness of rights-based RMC

Some but not all providers were aware of the actual document of MoPH’s Patients’ Charter of Rights, although distributed to all health facilities. All providers, however, asserted that women must be treated with respect and dignity during childbirth.

*“Yes, there is some chapter hanging outside on the wall and shows what the rights of the women are and that care is free”*

*When a woman attends the clinic we take her history, give her blood tests, respect her and keep her privacy and during giving birth we tell her about each step of care, if we inject her without telling this may be shocking to her*

### Suggestions for improvement of maternity care

Providers emphasized that ANC visits, birth preparedness and compliance of womens’ families with health advice and guidance will lead to better quality of the birthing process. Teamwork among providers when assisting childbirth was stressed to ensure uninterrupted and timely care to women and babies such as administration of medications and performing the required procedures. The providers expected government authorities, managing NGOs and facility administrations to recognize and reward providers for their good work. They should also ensure sufficient numbers of medicine, equipment, supplies, laboratory facilities, blood transfusion, ultrasonography and necessary power supply to make facility birth more acceptable to women. They expected to have senior doctors and surgeons to assist when needed and regular training and coaching to remain responsive to the women’s expectations.

*If there are no gloves, no suction, no equipment and clean item, it is not possible to work.*

*Well, different reasons may contribute [to disrespect], for example a midwife deliver five cases during the night duty and in the morning many patients are waiting, the midwife might be harsh because she is exhausted. More midwives should be hired.*

*We should be recognized for our hard work and rewarded with cash when we work better.*

### Experiences and expectations for respectful workplace environments

Providers deemed satisfaction of the women after birth at the time of discharge as “rewarding” and making them “happy”. When providers had to take care of multiple women simultaneously, they reported it as “stressful” when each woman demanded her not to leave her bedside. Providers reported being discouraged when supervisors fail to notice their hard work. Frequent night duties extending into full day shifts, excessive workload, lack of transport facilities to commute to their homes in uncomfortable times, low salaries, gaps in skills and confidence in performing certain tasks were reported as workplace stressors.

*It is rewarding to see a woman receive good care and leave the clinic healthy and happy. They pray for us and thank us which make us very happy.*

*When I first encountered a laceration - I only had experience with the model - and I was worried but I managed the case and was happy*

### C- Key informants’ perspectives

The key informants included twelve midwives and doctors (serving as clinicians and/or managers) with 2–28 years of experience working in central or provincial MoPH, seven BPHS representatives and one focal point from another sectoral ministry working on gender issues. They provided their viewpoints anonymously against open-ended iterative questions.

### Awareness of rights-based RMC

None of the respondents cited all nine elements of RMC as per the National Patients Charter. RMC was described in terms of kindness, lack of verbal or physical abuse, good behavior, privacy and respectful communication, with a few also mentioning no discrimination, provision of all available elements of care without delays.

*“For me, at least the provider should treat the woman kindly, listen to her, allow her to have a companion and maintain confidentiality”*

*“Respectful care is receiving all health care that all humans should receive and the human rights of women should be respected.”*

### Key informants’ narratives of maternity care, including quality of care and presence of mistreatment and abuse

The key informants admitted the presence of “rare” instances of verbal abuse (reprimanding women for having too many children, insulting, not listening) and physical mistreatment (slapping, pinching, beating and unnecessarily limiting women’s mobility) due to providers’ temper, discrimination against the poor and based on ethnicity. They also blamed shortage of supplies, lack of recognition mechanism for providers, low salaries and explicit absence of reference to RMC in the job descriptions of health providers.

*“Many of our women are Kuchi [nomads] and they are a little weak in their hygiene. With such women, [providers] behave extremely disrespectfully, and tell them: you smell bad, I don’t want to check you, get out of this room, go and wash your body first then come back”*

*“There are many reasons. The situation in the country [unrest and insecurity] causes all people to be tense and irritable. And when the health worker puts on a white gown she feels superior to others and she is tempted to be harsh and abusive to the women as they are in need of her care.”*

### Suggestions for ensuring RMC

Key informants pointed to the role of women and their families, healthcare providers, managing institutions and MoPH to make maternity care more respectful. Women were expected to attend ANC, comply with instructions, prepare in advance for childbirth, provide for medicines if not available and find a blood donor when needed. Providers were expected to work effectively as a team and provide all elements of care without interruption and delays. NGOs and the local MoPH officers were expected to recognize and reward health workers, ensure availability of equipment, supplies and medicines, electricity, laboratory and blood transfusion facilities, ultrasonography, training of providers and supporting them with employing surgeons and senior doctors.

*“The provider expects that the woman should be truthful and should not hide anything from the provider.”*

*“The woman should come for ANC. In many places the mother-in-law says we had 10, 12 children at home, why do you go to doctors so often? But they should come. If anything goes wrong, we can identify and help.”*

### Experiences and expectations for respectful workplace environments

Disrespect of midwives by doctors, work overload, low salary, lack of living facilities for providers near their facility were noted as workplace stresses. Solutions, such as hiring the husband of female doctors and midwives in some administrative or support capacity and recognizing the role of midwives were mentioned. They all emphasized the role of ANMC for quality midwifery education, recognition and accreditation of midwifery schools, continued education and providing a safe workplace for midwives.

*“The health [team] is teamwork in general. Having only a doctor, or only a midwife is not effective. When a woman comes [to hospital] she sees white gowns she does not differentiate who is doctor, who is midwife, who is nurse. She expects to be treated and taken care of by all. So the supervisor should make sure that all workers play their role. Someone should take the woman to the ward, someone should give her health education, even the guard and cleaner should guide her. Otherwise women will be discouraged and not come again.”*

## Discussion

Provision of healthcare in a respectful manner is an integral dimension of quality healthcare as recognized and formalized by WHO’s quality of care framework [9]. Mistreatment during maternity care, in any physical, behavioral or verbal disguise, is condemnable as violation of human rights and as a barrier to access care.

The women, providers and key informants confirmed a variety of mistreatment during provision of maternity care. Women understand the advantages of giving birth in healthcare centers in the presence of skilled birth attendants, but are concerned about violation of their dignity, which may inhibit seeking care. Reserved language used by some women such as reporting “some delays”, and stories about “other women” probably out of the fear of losing healthcare, indicates a latent dimension of the violation of their rights during receiving maternity care in facilities. This finding also raises the concern that RMC studies may not reveal the full scale and spectrum of mistreatment women face in maternity wards, thus undermining the attention required to address RMC.

Excusing providers for their abusive behavior towards women and justifying their harsh treatment, such as forcing certain birthing positions for women’s own benefit, hints to lack of women’s awareness of RMC and normalization of mistreatment in their behalf. Similar normalization of disrespect and abuse and excuses for mistreatment have been reported in other countries [23]

Discrimination based on relationship and socioeconomic status has been suspected and is confirmed by this study. Denial of care with excuses such as shortages of beds, attending after official working hours, attending at an early phase of giving birth, even if the woman comes from a long distance, demonstrate the immensity of inhumane treatment of women in some instances. The obstetricians, midwives and key informants in this study confirmed the various forms of mistreatment during maternity care, but they also acknowledged contributing factors such as shortages in staff, supplies, medicine, equipment and amenities, and other operational, environmental and cultural barriers. Other studies also implicated providers’ inadequate professional development and work dissatisfaction, poor systems and shortages could lead to less RMC during childbirth, insecurity, abusive working conditions and socio-cultural factors for less RMC [18, 24].

A key finding was the poor grasp of providers and senior health staff over the dimensions of RMC. Few respondents hinted to issues other than physical and verbal harm, missing aspects as freedom to ambulate, choosing birthing position, choosing and retaining companions, right to be informed about and consent to procedures to be carried out and right to stay in contact with the baby. Especially, lack of awareness about RMC policy documents shows how superficially the matter has been taken by providers and managers. Previous evidence also implicates lack of awareness about RMC among providers as a factor in mistreatment of women [19].

Since the study was conducted, the COVID-19 pandemic and associated hardships due to movement and contact restrictions, economic losses and diversion of resources are not good news for ensuring RMC.

Subsequently, in mid-2021, the collapse of the Government in the hands of Taliban with little regard to human rights effectively banished women from social life and the significant progress of the past two decades was reversed [25]. A systematic and ever accelerating drainage of human capital from Afghanistan and drastic drop in external technical and material assistance were the tragic results. Systematic cancelation of women’s rights to freedom of movement, losing autonomy to male guardians, limited options for jobs, restrictions on education and career building are daunting realities [26, 27]. Nevertheless, Afghan women continue to give their lives to the future generation and remain as direly as ever in desperate need for respectful, dignified high quality care. Therefore, the conclusions and recommendations of this study can be useful to the de facto Afghanistan health authorities, national and international technical partners and the donors to ensure maternity care is provided respectfully to Afghan women.

### Recommendations

Lack of awareness and knowledge of RMC was well established in the findings. Therefore, social and behavioral change and communication (SBCC) using mass media, education in health facilities, community events, social fora including religious sessions of Friday and Eid prayers, and public addresses by health authorities about RMC are essential to address lack of awareness. Such awareness promotion has been found a key intervention by another study [28]. On the other hand, improving provider’s side is equally important. Building the capacity of providers through training in RMC has been shown to be significantly associated with improved quality of care [29]. Therefore, training, mentorship, coaching and peer learning of healthcare providers in RMC, during pre-service and in-service periods using tools such as the International Confederation of Midwives’ (ICM) RESPECT toolkit are recommended [30]. SBCC activities will also be needed to institutionalize the change among the providers’ community. Measures to address the identified RMC-gaps, such as mistreatment, denial of companions, restriction of birthing positions and normalization of such violations, need to be enforced. In the face of the Taliban’s rule, it is important that RMC is valued and monitored by donor agencies and those providing technical and financial aid to the health sector. For example, women’s satisfaction with care as a core component of quality care. Monitoring of RMC is therefore important and indicators measuring the level of adherence of providers to RMC requirements can be adapted from existing sources and integrated into national monitoring tools [31]. This integration may include existing tools such as WHO’s labor care guide that help ensuring labor companionship, pain relief and women’s comfort [32]. For example, USAID’s statement of support for healthcare in Afghanistan emphasizes women’s rights while the World Bank commits to financing basic healthcare [33, 34].

### Limitations

The study was conducted in 2018 under relatively stable conditions, before the devastating effects of the COVID-19 pandemic in 2019 and the regressive reset in health and development due to the change of Government in 2021. Therefore, the current situation of maternity care might be worse. The study was conducted in larger provinces for security and accessibility reasons. However, this may not be representative for all different populations in the 34 provinces of Afghanistan. On the other hand, while selection of the semi-rural DH facilities was considered relatively representative of both rural and urban settings, there could be subtle differences with smaller rural facilities. Therefore, our findings need to be interpreted with caution to other regions and provinces of Afghanistan.

Other issues documented during this study were out of pocket expenses imposed on families and problems between healthcare providers in health facilities. The ability of families to give tips and gifts may determine how RMC is provided. Interpersonal issues among healthcare providers may also lead to retaliatory disrespect towards those who receive maternity care.

## Conclusion

Lack of awareness of the increasingly disempowered and sidelined Afghan women about their right to RMC and poor knowledge and attitude of providers about rights-based RMC are major issues that need to be addressed by appropriate awareness and capacity building initiatives. Finally, the degrading human rights conditions in Afghanistan call for a set of powerful responses from civil society, independent institutions and international organizations such as AMA and AFSOG, ICM, MSF, WRA, United Nations (UN) and other bilateral and multilateral organizations. These may include pro-women and pro- RMC position statements, frequent diplomatic discourses, resolutions, financial commitments and proactive technical assistance rather than nonconsequential verbal condemnation of the situation.

### Supplementary Information

**Additional file 1.**

## Data Availability

Data are available from Jhpiego upon reasonable request and signature of a data sharing agreement. Requests should be directed to Hibest Assefa at OpenDataHelp@jhpiego.org or Hannah Tappis at Hannah.Tappis@jhpiego.org.

## Abbreviations

AFSOG: Afghanistan Society of Obstetricians/Gynecologists
AMA: Afghan Midwives Association
AMC: Afghanistan Medical Council
ANMC: Afghanistan Nursing and Midwifery Council
BPHS: Basic Package of Health Services
CME: Community Midwifery Education
DH: District Hospital
EmONC: Emergency Obstetric and Newborn Care
EPHS: Essential Package of Hospital Services
FGD: Focus group discussions
ICM: International Confederation of Midwives
IHS: Institute of Health Sciences
IRB: Institutional review board
JHBSPH: Johns Hopkins Bloomberg School of Public Health
KII: Key informant interview
MCHIP: Maternal and Child Health Integrated Program
MOPH: Ministry of Public Health
NGO: Non-governmental organizations
SBCC: Social and behavioral change and communication
UN: United Nations
USAID: United States Agency for International Development
USD: United States Dollar
WHO: World Health Organization
WRA: White Ribbon Alliance
