# Supplementary material for: Women’s experience of childbirth care in health facilities: a qualitative assessment of respectful maternity care in Afghanistan
Source: BMC Pregnancy Childbirth. 2024 Jan 10;24:48. doi: 10.1186/s12884-023-06234-9 (PMC10777596; doi:10.1186/s12884-023-06234-9)
Supplement: Supplementary file 1 — Additional file 1. [file 12884_2023_6234_MOESM1_ESM.docx]

**Women’s experience of childbirth care in health facilities: a qualitative assessment of respectful maternity care in Afghanistan**

**Supplementary Annex**

Illustrative quotes from the study respondents on thematic areas

| **A-Women´s perspectives** |
| --- |
| A1-Motivators for using health facilities for childbirth |
| - - “Well, God forbid, if the baby is in wrong position or bleeding happens, or [baby] is weak, there are midwives in the clinic and they will give you injections or pills or they will send you to another hospital for treatment.”   - “Everyone treats us nicely, they check my blood pressure, give me serum, talk to me nicely”.   - “The doctors know (what to do), the Dais and old women know nothing, I will not let them touch me”.   - “Illiterate women deliver at home…Hospital was a comfort”.   - “In the clinic they look [examine] carefully, give drugs and serum in case you have bleeding or high blood pressure, take the baby [deliver] cleanly and wrap the baby properly, there are midwives and doctor”.   - “They [healthcare providers] treat us nicely and take care of us”.   - “They [women] come to hospital for cleanliness, knowledgeable people and midwives, their privacy is respected, only one companion like mother of mother-in-law or sister is allowed. Some women that are illiterate say “we don’t go to hospital, out privacy is not maintained” but the women who know say “we go to hospital because if something is wrong like bleeding, it is better to be in hospital” |
| A2-Deterrents from using health facilities for childbirth |
| - - “Old people [women] told us we always delivered at home, you youngsters are weak and afraid of giving birth . … [therefore in past] I was ashamed on the midwives… but when I came, it was a big comfort to deliver in health facility.”   - “Some [people] have no money to come to clinic; they have to come from a large distance”   - “Women want to come to hospital [for giving birth] but husbands think it is a shame”   - “The woman should not be offended by neglect of the providers in the facility, because no one pays attention to them they will not come to hospital and will deliver at home”   - “Some families are not open [conservative], so even I remember one of my neighbors had contractions for 4 or 5 nights but she always said that I am ashamed to go to the clinic. My family told me that “you are going to facility, you aren`t shamed to go there?”   - “Some people think that home birth is honorable. Some women think that in old times all women delivered at hom and nothing happened [to them], you [women] should also deliver at home”   - “My husband was telling me to deliver at home as he could not afford the hospital, but my mother-in-law quarreled with him and reprimanded him.” |
| A3-Women’s experiences of providers’ behavior (including proper and mistreatment) |
| - - “The hospital was clean, the aunty [cleaner] was also very clean. I felt safe inside.”   - “They [healthcare provider] stay with me, gave me serum and injections, checked my blood pressure. They looked at their watch and when the time was up they told me to push…finally the baby came, they put the baby on my belly… they watched me and my baby for six hours…then they took my phone number and said she will call me sometimes….if anything happens call me or come back”.   - “She [provider] treated me nicely. When I came she gave me serum and injection and told me to wait. She was watching a serial drama in the TV, my mother-in-law went to her and told her that I am in pain, she came and checked me and said with smile that I still have time”   - “We came [to hospital] at one o’clock of night [1am???], [she] helped me and gave me prescription, told me there is still time and go back home…when I came home I delivered at home with my mother-in-law. She did not give me any medicine and I went back home.”   - “Of course they treated me badly, if they didn’t why they didn’t help me [ignored me]? They discriminate between wealthy and poor. They accuse us that we linger in the facility unnecessarily and we try to find excuses to stay in hospital”.   - “The aunty [a term used for cleaner in respect] helped me more. The doctor does not see [examine] me properly. When I have trouble I got to aunty [cleaner]”   - “I went to the hospital and midwife examined me (vaginal) and said still you have time so I went to washroom then my child was born there. Even I had the same experience with my second child. Midwife said ‘do not say to anybody that you went outside the giving birth room’. “   - “All roads were closed so with walking through streets we came to the hospital. The long walk increased my bleeding. When I arrived at the clinic they said we do not have any space for you because we have many women right now. They didn`t accept me and I walked back home ...”   - “Midwife said to woman’s companion that our official working time is till 4 so if there were no real contractions or any progress, I will send you back home. The companion begged for the woman to stay longer in the hospital. As he said - I only have 1000 Afghani to go back home [it is not enough to return back to the hospital again] ”   - “One woman came from very far away, and she gave birth in the car. But her placenta was not delivered at the time of her arrival to the hospital. Hospital midwives helped us to deliver our babies but none of them helped that woman because they were angry - why did the woman deliver in the car?”   - “Midwife does not understand the pain that we have in giving birth. She used her hand to pick up the newborn and hit and shouted at me to spread your legs”   - “[I delivered] in the clinic, [they] look at me [take care of me] properly, treat me, give me serum and injection, take care of bleeding, issues in blood pressure, high or low, take the baby clean and in orderly manner and clothe the baby [properly]”   - That night three women delivered and one midwife was there for each. One midwife was standing near to me I was afraid, I was trembling, only one midwife was there, I was worried what if something goes wrong with me, but had no problem. … Midwife conducted a little harsh but it must be difficult for them too, I have no complaints. We were in one room, only a thin curtain was between us, the cleaner and my mother were present, but I understand, I have no complaint.   - “Specifically, they behave badly with the poor”   - “Certain doctors that don’t have experience behave badly, [those] that have experience they are good, younger [doctors] are inexperienced” |
| A4-Expected behavior of care providers |
| - - “They [providers] help, when you have pain they help, you feel reassured that here you have midwife she will help if anything happens”   - “A doctor should have good behavior, should give us good morale during giving birth, should not reprimand us for anything.”   - “The midwife would not come to my bed unless we insisted repeatedly, and finally she came when I was about to deliver.”   - “When I came in the night, my mother and mother-in-law were with me. In the morning it was more crowded and the doctor told us that only one person can stay with me.”   - “They told my companion not to come in” |
| A5- Awareness of right-based RMC |
| - - We have the right, that we come here at the time of illness [giving birth], we must be treated here, this is our right]   - “It is written that everything is free, so we come, if it were for money, we would not come”   - Our right is that we come here for treatment, doctor takes care of us, we give birth to our child and we go home happily”   - “No one is aware of these rights! If it is printed [written] only those who can read know. They should tell those who are illiterate about their rights”   - “By God, I was not aware [of right to services] |
| A6-Out-of-pocket payments |
| - - “This time, the doctors told me that I will need Cesarean [section], and when I delivered normally they told me I should give them “treat” [gift in cash] so I give them five hundred Afghani as gift.”   - “My husband’s sister gave birth here, he was a boy, the doctor asked for 500 Afghani, we gave her money. Everybody asks for presents; the cleaner asks for presents as well”   - “Last time when I went to the hospital midwife and doctor said without our help you might have CS so we do not know [for this help] you will pay Shirini (sweets or gifts) or not. I said why not, I paid 500 Afghani as shirini”   - “We thought if we pay shirini then we will receive much better care.”   - “Sometimes Dai are not happy, they tell us for money, they say don’t go to clinic …. If you deliver here [with dai] it will be 500 or 600 [Afghani] but if you go to facility, [although] they say it is free but you will have to spend 2000 or 3000 [Afghani]. Stay here, spend only 500”   - No, [they provide care] without money, this hospital is free”   - Always the midwives tell us to come to hospital for giving birth…. Everything is free”   - This [last] time, when I came to hospital, the midwife told me you should have done Cesarean Section, now that you did not, should you not give gifts? I said yes why not, I will give gifts. Then I gave them 500 [Afghani]; 300 to the midwife and 100 to [two] cleaners. I gave it myself [voluntarily]. Midwife complained 300 is little [amount]”   - “We say, if we give them gifts, they will take better care of us”   - My children’s father [husband] said, had I given them the gifts sooner they would have taken even better care of me”   - “Oh, yes, when I gave birth to these three children of mine, doctors took 500 and cleaners took 200 each”   - “they say bring [give] the money, or else they will not write the prescription for us” |
| A7-Suggestions for improvement of maternity care |
| - - Number of [birth] rooms need to be increased, birth rooms should be warm, in the summer [rooms] should be cool.   - “One woman said that electricity should come, another one that ultrasound should come”   - They should observe the queue, when we come two persons from one family, they tell us chose which one should be examined [and treated], because they can take care of only one person from one family”   - We want that a knowledgeable doctor should come to help us better. They should take care of rich and poor alike. They give only four [very few] tablets to the poor, we wait from morning to afternoon but they don’t give us medicine, don’t help us” |
| **B- Providers’ perspectives** |
| B1-Motivator for use of health facility |
| - - “The death and life is in the hand of Allah, however, every woman wants to give birth in a clean and safe place where skilled birth attendant is available to provide all of the services”   - “A woman who attends the facility for four antenatal visits, gets medicine for anemia and receives health education and counselling on vaccination, birth planning, checking blood group and checking anemia. Therefore, such women come to clinic for giving birth” |
| B2- Deterrents for use of health facilities |
| - - “Just because of this poverty they can’t come to facility. Some [of them] come from a distant [place] and if they want to come they don’t have the money for transportation. In the middle of the night, a car will ask at least two thousand Afghanis. Also the people’s level of knowledge is low, and there is insecurity too.”   - “One of the reasons is the [bad] behavior of the midwives because of too much work. The woman says I will not go there [to the clinic] unless I have a problem after my birth at home. Then if she [the woman] comes to hospital and for example has retained placenta, if the midwife can’t help her [immediately] in the birth room, she will need to stay in hospital for two three days, she should get antibiotics, misoprostol, get her uterus emptied, therefore when we say come to clinic she [the woman] says she will prefer to die at home but not come to hospital.”   - “One woman came when she was at risk of uterus rupture. When asked why you came late, she said my sister in law said that ‘she is showing off …. I had between 10-15 births but till my 5th birth I never seen any doctor or midwife. Why you are going to clinic?”   - “We also have this idea, that most [women] have complaints that their mother-in-law does not allow their husbands to take them to clinic. They [husbands] bring the women only when she has complications and her condition is worsening.”   - “Most of the women who have pre-eclampsia or eclampsia, their mother-in-law will not allow them [husband] to take them to clinic and will force them [husband] to take them [woman] to mulla [local religious leader] |
| B3- Providers’ narratives of maternity care including quality of care and presence of mistreatment and abuse |
| - - “Our birth room is clean and we have a separate postpartum room with two beds. The door has a curtain, we change the bedsheets and allow the woman to give birth the way she likes. Some women even don’t allow their mother, but if she likes we allow companions to stay with her during giving birth. Some husbands bring their wives on a bike, so we have to deliver her alone without someone from her family. Women can walk, eat, drink as they like until the cervix is dilated enough. We insist that women should give birth on the birth table because it is clean, but if the woman cries and makes noise [insists very much], we spread a plastic sheet on the floor and allow her to give birth on the ground.”   - “Some women request to not allow their mother in law to accompany her. She said please tell her we only allow one person or we are not allowing any companion. If any complication happened, we will inform you. Women said then they [mother in law] will scoff at us -which position you delivered, who saw your body”   - “First of all we introduce ourselves, we don’t address the women as “aunty”, or the sort, but call them by name. We speak in medical terms between ourselves but then explain to the woman in easy language so they will not be worried. We listen to women. If one woman goes out of the hospital with no satisfaction, more patents will not come.”   - “I have seen with my own eye, not here, in another clinic that the doctors beat women, they [women] are in pain but they [doctors] give them [women] no medicine”   - “We have seen that the elders have complained [to the hospital manager] that their women are not treated well, they are not given serum, pills, injections, and their blood pressure is not checked   - Many of women are Kuchi [nomads] and they are a little weak in their hygiene. With such women, [providers] behave extremely disrespectfully, and tell them: you smell bad, I don’t want to check you, get out of this room, go and wash your body first then come back   - No I personally try to be nice with my women because I am also a mother I never do anything to hurt a woman during her pains. And I try to help her treat her nicely encourage, but there are women who kicked me when I tried to examine and later she apologized. Sometimes when the woman or the companion demands too much interferes with treatment, insist on injection and serum, a midwife who has delivered ten - eleven women at night duty may react and be harsh naturally have heard such complaints from families that the provider had bad behavior with the women. In my 15 years’ experience I can’t say I have not seen such cases |
| B4- Providers’ experience of out-of-pocket payment for maternity care |
| - - “The other day, a woman had a boy after four daughters. Next day she sent me some dried spinach and insisted that I should take it. If I did not take it she would be offended.”   - Till now all the medicine is free, but they [women and families] give us gifts.   - Some women give us 100 or 200 Afghani when their baby is born, God forbid, we never ask for money. People are very poor, very poor”   - Once an uncle [senior man] who was with a woman that I helped told me offered me some money, I refused and told him that we have salary and all services are free, but the uncle insisted and told me that if I don’t accept his gift he will be offended. I said please take your money, buy some fruit for your woman. The man was militant and had turban, so I had to accept his gift”   - I have salary, I have perdiem, I have no right to take any money from people, all services are free here.   - The intern told me” Doctor you must take some gifts, she instead until I had to get gift from my uncle’s granddaughter who give birth in our clinic. |
| B5- Understanding/awareness of right-based RMC |
| - - Yes, there is some chapter hanging outside on the wall and shows what the rights of the women are and that care is free   - When a woman attends the clinic we take her history, give her blood tests, respect her and keep her privacy and during giving birth we tell her about each step of care, if we inject her without telling this may be shocking to her   - I think we need to improve our capacity, [RMC] should be made part of curriculum and medical ethics should be taught in institutes and school. |
| B6- Suggestions for improvement of maternity care |
| - If there are no gloves, no suction, no equipment and clean item, it is not possible to work   - Well, different reasons may contribute [to disrespect], for example a midwife deliver five cases during the night duty and in the morning many women are waiting, the midwife might be harsh because she is exhausted. More midwives should be hired   - There should be equipment and tools, a standard room, 24-hour electricity, water, timely supply of medicine… if not, people can’t benefit from the facility perfectly   - Our expectation is that every month or two, they should invite us for trainings to improve our knowledge, we don’t have city power, only solar power that in winter it does not work   - We should be recognized for our hard work and rewarded with cash when we work better. |
| B6- Experiences and expectations for respectful workplace environments |
| - - It is rewarding to see a woman receive good care and leave the clinic healthy and happy. They pray for us and thank us which make us very happy.   - When I first encountered a laceration - I only had experience with the model - and I was worried but I managed the case and was happy   - I can say in past one and a half years I had no complaints against midwives, the society is more involved and even we share our budget with people   - I have heard of a case that the woman slapped the doctor, we talked to the community and the man apologized |
| **C- Key informants’ perspectives** |
| C1-Awareness of right-based RMC |
| - - “For me, at least the provider should treat the woman kindly, listen to her, allow her to have a companion and maintain confidentiality”   - “Respectful care is receiving all health care that all humans should receive and the human rights of women should be respected.”   - “We should inform all families and people about their right to health services. All health workers from the guards to cleaners to providers should be educated about the woman rights; there should be a monitoring system that everyone complies with the requirements. If there is any flaw in the way they [providers] treat the woman, they should be trained and encouraged to be respectful.” |
| C2-Key informants’ narratives of maternity care, including quality of care and presence of mistreatment and abuse: |
| - - “Well, the care is respectful, definitively respectful, however maybe not a hundred percent of times… for example when we see that a doctor or midwife examines a woman in an inappropriate location, where the privacy is not maintained, and ask why she does not take her to a place where her privacy could be maintained… they say we have no place, all the rooms and beds are occupied, it was the only place that I had.”   - “There are many reasons. The situation in the country [unrest and insecurity] causes all people to be tense and irritable. And when the health worker puts on a white gown she thinks she is superior to others and she is tempted to be harsh and abusive to the women as they are in need of her services.”   - “I have heard many stories but have not seen it. When a woman has many children, sometimes with low voice, sometimes louder, the provider advises her to stop and bring no more children.”   - “I heard that a doctor had beaten a woman. And another one pushed a woman [disrespectfully].”   - “I don’t want to specify, but long ago a person with white gown next to the woman slapped the woman hard and used inappropriate words that I can’t repeat here. I could not defend her because I was not a doctor [yet], but if there was a system and any health worker who abuses a woman is disciplined, such events will not be repeated.” |
| C3-Suggestions for improvement and ensuring RMC |
| - - “The provider expects that the woman should be truthful and should not hide anything from the provider.”   - “The woman should come for ANC. In many places the mother-in-law says we had 10, 12 children at home, why do you go to doctors so often? But they should come. If anything goes wrong, we can identify and help.”   - “Sometimes the providers cannot be blamed. The women have no hygiene and that upsets the provider. We expect that women also take care of their hygiene.” |
| C4- Experiences and expectations for respectful workplace environments |
| - “I have worked in a clinic; if you want to be effective as a clinician you have to work as a whole team.”   - “The health [team] is teamwork in general. Having only a doctor, or only a midwife is not effective. When a woman comes [to hospital] she sees white gowns she does not differentiate who is doctor, who is midwife, who is nurse. She expects to be treated and taken care of by all. So the supervisor should make sure that all workers play their role. Someone should take the woman to the respective ward, someone should give her health education, even the guard and cleaner should guide her. Otherwise the women will be discouraged and will not come again.”   - “The supervisor should be kind and mind [her] language with the providers. If the supervisor is angry the provider will be angry with the woman.”   - “There are issues that the leaders should know. The workers that do their jobs correctly should be recognized and rewarded. This enhances the capacity of the providers. They should praise those who perform better.”   - “MoPH should treat all health workers [equally]. It is not correct that one provider working in a remote area is forgotten and no one knocks on her door, so if the visitors visit her she will be very motivated.” |
